# Diagnosis of Rare Cervical Tumors: IECC Classification, HPV Status, and Subtype-Directed Immunohistochemistry, with Treatment Outcomes from a Tertiary Center

**DOI:** 10.3390/pathogens15090907

**Published:** 2026-08-28

**Authors:** Osman Doğan, Mürşide Çevikoğlu Kıllı, Mehmet Sait Bakır, Şahin Yüksek, Duygu Kolukısa, Numan Bilgiç, Hasan Turan

**Affiliations:** 1 Department of Gynecologic Oncology, Mersin City Research and Training Hospital, Mersin 33240, Türkiye; 2 Department of Pathology, Mersin City Research and Training Hospital, Mersin 33240, Türkiye; dduygukuru@gmail.com

**Keywords:** rare cervical tumors, diagnostic pathology, immunohistochemistry, IECC classification, HPV-independent cervical cancer, p16, case series

## Abstract

**Background/Objectives:** Rare histologic subtypes of cervical cancer are uncommon and may be misclassified during initial diagnosis. Their recognition depends on subtype-directed immunohistochemistry, which excludes metastatic mimics, and on high-risk HPV (hrHPV) testing, which establishes HPV status. Published series rarely report how the diagnosis was reached. We therefore documented the full diagnostic work-up, together with treatment and outcomes, in a consecutive single-center series. **Methods:** This was a retrospective, single-center case series (January 2020–January 2025). Rare tumors were defined per the 2020 WHO Classification as subtypes other than squamous cell carcinoma and usual-type adenocarcinoma. Immunohistochemistry was used to confirm lineage and exclude mimics. hrHPV DNA testing and p16 immunohistochemistry were performed where tissue permitted. A tumor was called HPV-associated only when diffuse block-type p16 positivity accompanied hrHPV DNA; HPV DNA alone was not accepted as evidence of an HPV-driven tumor. **Results:** Ten patients were included (median age 53 years). There were two signet-ring cell adenocarcinomas, two small cell neuroendocrine carcinomas (SCNECC), two serous adenocarcinomas, and one each of sarcomatoid carcinoma, clear cell carcinoma, granulocytic sarcoma, and poorly differentiated adenosquamous (glassy cell) carcinoma. hrHPV DNA was detected in six of the seven tumors tested. Three of these six were p16-negative: one serous, the clear cell, and the glassy cell carcinoma. Because diffuse block-type p16 expression is a validated surrogate marker of transcriptionally active hrHPV, we classified these three as HPV-independent. This matches the known biology of these histotypes. Lymphovascular space invasion was present in eight patients (80%). Over a median follow-up of 10.5 months, four patients developed distant metastases and three died. **Conclusions:** Accurate diagnosis of rare cervical tumors requires subtype-directed immunohistochemistry to exclude mimics and hrHPV testing interpreted together with p16; HPV DNA positivity alone does not establish HPV-association. Given the small, heterogeneous series, outcome data are descriptive and hypothesis-generating. We propose a diagnostic algorithm and immunohistochemical framework and advocate centralized review and prospective registries.

## 1. Introduction

Squamous cell carcinoma (SCC) accounts for approximately 75–80% of cervical cancers and is strongly associated with HPV infection. Endocervical adenocarcinoma represents about 20–25% of cases, with 10–15% occurring independently of high-risk HPV. Rare histologic subtypes include neuroendocrine carcinoma (0.9–1.1%) and glassy cell and gastric-type adenocarcinomas (approximately 3%), which are generally HPV-independent and associated with poorer prognosis [1,2]. Because each subtype is uncommon, it is easily misdiagnosed. Histology alone is not enough. Immunohistochemistry is needed to confirm lineage and to exclude metastasis from an extragenital primary, and HPV testing is needed to establish the tumor’s relationship to the virus.

Persistent high-risk HPV (hrHPV) infection is the necessary cause of most cervical cancers [3]. A few oncogenic types, mainly HPV16 and HPV18, account for the majority, and their relative contribution differs by histology [4]. The IECC system and the 2020 WHO Classification group cervical tumors by causation into HPV-associated (HPVA) and HPV-independent (HPVI) categories [1,5]. This distinction matters clinically: HPVI adenocarcinomas have worse disease-specific survival than HPVA tumors [6], and HPV-negative cervical carcinoma is a distinct, prognostically unfavorable entity [7]. Assigning HPV status therefore requires more than morphology. Diffuse block-type p16 expression is used as a surrogate for transcriptionally active hrHPV, whereas HPV DNA alone does not establish an HPV-driven tumor [8].

The expected HPV relationship differs by subtype. SCNECC is usually HPVA and needs neuroendocrine markers for confirmation because morphology is unreliable [9,10,11]. Glassy cell carcinoma is usually HPVA but may be HPV-negative; the 2020 WHO Classification regards it as a poorly differentiated adenosquamous carcinoma rather than a separate entity [12,13]. Sarcomatoid carcinoma is HPVI and is distinguished from a true sarcoma by focal epithelial differentiation [14]. Signet-ring cell carcinoma requires exclusion of gastrointestinal metastasis before a cervical origin is accepted [15,16]. Clear cell and serous carcinomas are traditionally HPVI. Molecular studies confirm that HPV-independent cervical adenocarcinomas form a distinct group with characteristic mutations and a worse prognosis [17]. Primary cervical granulocytic (myeloid) sarcoma lies outside the HPV framework and is treated systemically [18].

Published series of rare cervical tumors seldom report how the diagnosis was reached: which antibodies were applied, which mimics were excluded, which HPV method was used, and on what basis HPV status was assigned. Readers therefore cannot judge how securely a diagnosis was established. We address this gap by documenting the full diagnostic work-up in a consecutive single-center series and by proposing a diagnostic algorithm and an immunohistochemical framework.

## 2. Materials and Methods

### 2.1. Study Design and Patients

This retrospective case series was conducted at the Department of Gynecologic Oncology, Mersin City Research and Training Hospital, between January 2020 and January 2025. We included consecutive patients with a rare cervical tumor, defined per the 2020 WHO Classification [1] as any subtype other than squamous cell carcinoma and usual-type endocervical adenocarcinoma. Tumors that represented metastatic spread from an extragenital primary were excluded after clinical, endoscopic, radiologic, and immunohistochemical evaluation. The series is small and histologically heterogeneous, so no statistical analysis was performed. The findings are descriptive, and continuous variables are given as median (range).

### 2.2. HPV Detection, Genotyping, and p16 Assessment

hrHPV DNA testing was performed on formalin-fixed, paraffin-embedded tumor tissue in the Mersin City Hospital virology laboratory. We used a Qiagen real-time PCR assay (QIAsymphony RGQ System Qiagen, Hilden, Germany; Cat. No. 9001850). The assay identifies HPV16 and HPV18 separately and reports the remaining high-risk types together as “other high-risk HPV”. Results were recorded as HPV16, HPV18, other hrHPV, not detected, or not tested. p16 immunohistochemistry (clone BC42) was categorized as diffuse block-type positive (defined as diffuse, continuous nuclear and cytoplasmic staining), focal/positive, or negative, and was interpreted as a surrogate marker of transcriptionally active hrHPV when showing a diffuse block-type pattern. HPV status was then assigned by four rules. Tumors with diffuse block-type p16 positivity and hrHPV DNA were called HPV-associated. Tumors without hrHPV DNA were called HPV-independent. Tumors with hrHPV DNA but negative p16 were also called HPV-independent, because viral DNA without diffuse block-type p16 expression does not demonstrate transcriptionally active infection [8,17]. Non-epithelial tumors fall outside the HPV framework, and untested tumors were left unclassified.

### 2.3. Immunohistochemistry

**Figure 1:**
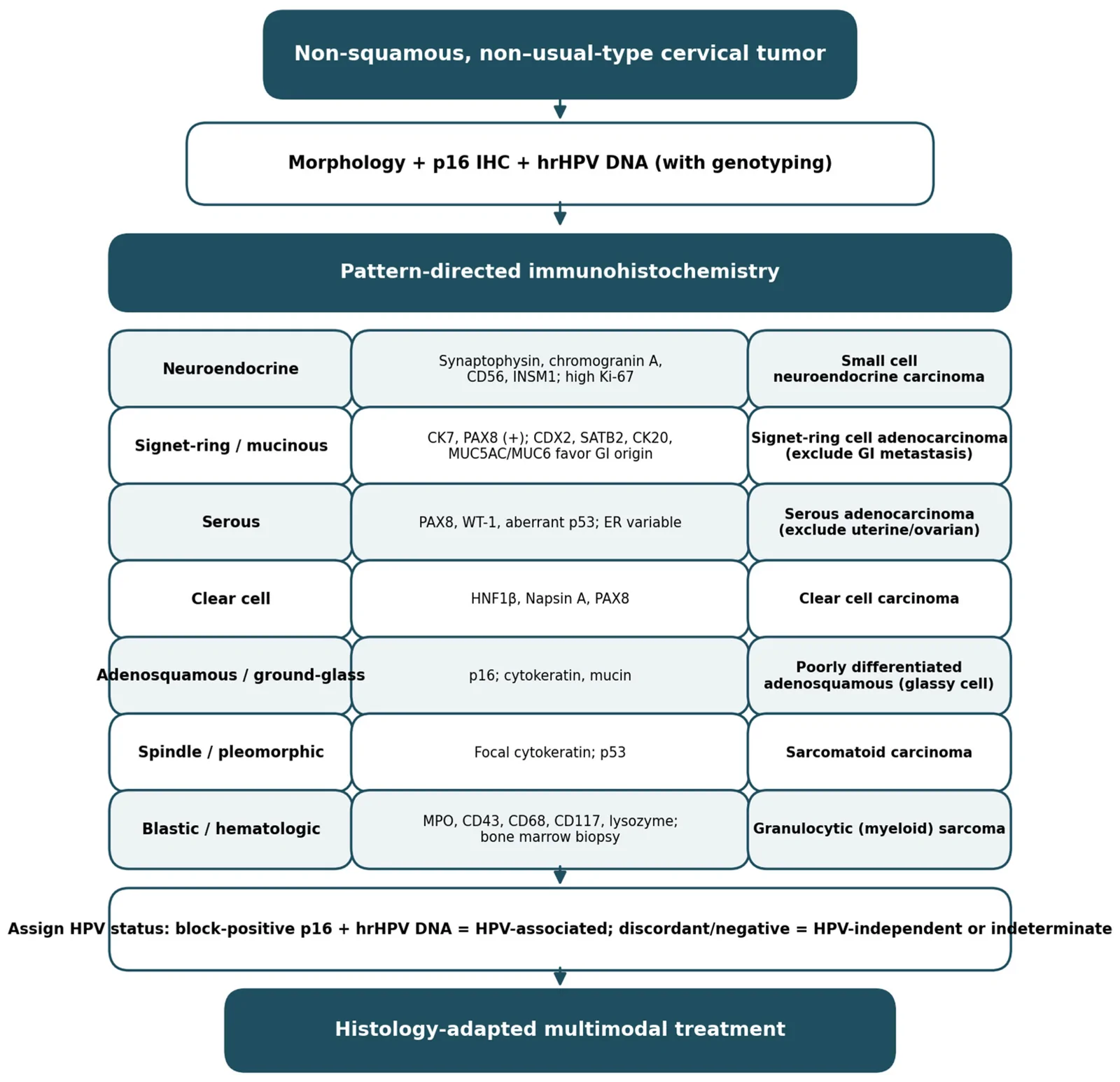
Proposed diagnostic algorithm for rare cervical tumors (schematic; does not depict patient data). Morphology is combined with p16 immunohistochemistry and hrHPV DNA testing with genotyping; pattern-directed immunohistochemistry then confirms lineage and excludes metastatic mimics. HPV status is assigned from p16 and HPV DNA read together—diffuse block-type p16 positivity with hrHPV DNA indicates HPV-association, whereas a p16-negative result (including hrHPV DNA-positive/p16-negative tumors) indicates HPV-independent status—after which histology-adapted treatment is selected. GI, gastrointestinal; hrHPV, high-risk human papillomavirus; IHC, immunohistochemistry.

Immunohistochemistry was performed on 4-µm sections of formalin-fixed, paraffin-embedded tissue. We used an automated platform (TALENTTM Automatic IHC Stainer, Xiamen Talent Biomedical Technology Co., Ltd., Beijing, China) with heat-induced epitope retrieval by using either citrate buffer (pH 6.0) or EDTA buffer (pH8.0) depending on the antibody. We followed the manufacturer’s protocols including positive and negative controls. Immunostaining was performed by using a poly-HRP-based detection system). Antibody clones, vendors, dilutions and retrieval tamponade are listed in Supplementary Table S2. The reporting pathologist selected the panel for each case according to its differential diagnosis. Not every marker was therefore assessed in every case, and a marker not reported for a case was not performed rather than negative. The resulting subtype-directed diagnostic algorithm is summarized in Figure 1.

### 2.4. Data Collection

We collected four groups of variables. Demographic and clinical data were age, presenting symptoms, smoking history, and ECOG performance status. Pathological data were cervical cytology, histologic subtype, tumor size, lymphovascular space invasion (LVSI), surgical margin and lymph node status, and FIGO 2018 stage [19]. Virological data were hrHPV status and genotype, p16 result, and HPV status. Treatment and outcome data were the treatment given, chemotherapy regimens, site and date of progression or metastasis, salvage therapy, and date of last follow-up or death. Bone marrow biopsy was recorded for the patient with granulocytic sarcoma.

### 2.5. Treatment

Treatment was decided by a multidisciplinary tumor board. Surgery comprised radical hysterectomy with pelvic ± para-aortic lymphadenectomy. Concurrent cisplatin (40 mg/m^2^ weekly) was the radiosensitizer in all patients receiving chemoradiotherapy (CRT). Systemic regimens were subtype-adapted: cisplatin–etoposide for SCNECC; carboplatin–paclitaxel ± bevacizumab for advanced signet-ring cell and serous adenocarcinoma; capecitabine–oxaliplatin for recurrent HPV-independent signet-ring cell carcinoma; and cytarabine–daunorubicin (AML induction) for granulocytic sarcoma. Interstitial brachytherapy was used in one patient with a bulky tumor.

### 2.6. Follow-Up and Outcome Definitions

Progression-free survival (PFS) was the interval from initial treatment to documented disease progression (local recurrence or distant metastasis), with censoring at the last follow-up. Overall survival (OS) was the interval from diagnosis to death from any cause, censored at the last follow-up. Progression required radiologic or pathologic confirmation. Because only three deaths and four progression events occurred among ten patients, the median OS and PFS were not reached; follow-up is reported as the median (range) of observed times.

### 2.7. Ethics

The study was approved by the Ethics Committee of Mersin City Research and Training Hospital (Approval No. 170; 25 March 2026) and followed the Declaration of Helsinki. Written informed consent was obtained through the institution’s standard procedure from all patients or from the next of kin where the patient had died. Consent covered the use and publication of anonymized clinical, pathological, and outcome data. No patient identifiers or initials appear in the manuscript or Supplementary Materials.

## 3. Results

### 3.1. Patient and Tumor Characteristics

Ten patients were included (median age 53 years, range 33–82). Vaginal bleeding was the commonest symptom (six patients), followed by discharge (two), abdominal pain (one), and distension (one). Three patients smoked. Cytology showed HSIL in three patients, ASC-H in one, AGC in one, invasive carcinoma in one, and normal cytology in one; cytology was unavailable in three. By FIGO 2018 stage [19], four patients had stage I disease, two stage II, three stage III, and one stage IV. There were two signet-ring cell adenocarcinomas, two SCNECC, two serous adenocarcinomas, and one each of sarcomatoid carcinoma, clear cell carcinoma, granulocytic sarcoma, and poorly differentiated adenosquamous (glassy cell) carcinoma. LVSI was present in eight patients (80%); the two LVSI-negative patients had clear cell and glassy cell carcinoma. Median tumor size was 4.1 cm (range 2.4–7.0). Full characteristics are in Table 1. Additional de-identified case-level clinicopathological details are provided in Supplementary Table S1.

### 3.2. Immunohistochemical Findings

The stains performed in each case and their results are given in Table 2. Diagnostic lineage was supported immunohistochemically in most cases. The signet-ring cell tumors were CK7-positive with CDX2 and SATB2 negativity, PAX8 negativity in Case 2, and MUC5AC positivity with MUC6 negativity. The clear cell carcinoma was HNF1β-, Napsin A-, and PAX8-positive. The granulocytic sarcoma expressed MPO, CD43, CD68, CD117, and lysozyme with CD20/CD3 negativity, confirming myeloid lineage and excluding lymphoma. The sarcomatoid carcinoma showed cytokeratin, EMA, vimentin, and mutant-pattern p53 expression, with focal epithelial differentiation distinguishing it from a true sarcoma. Two diagnoses warranted additional pathological review. In Case 7, classified as SCNECC, synaptophysin and CD56 were negative, chromogranin A was positive, INSM1 was equivocal, and Ki-67 was 30-40%, while p63 and CD99 were positive. The reporting pathologist re-reviewed the case with unchanged control staining, and the diagnosis of small cell neuroendocrine carcinoma was retained on the basis of characteristic small-cell morphology and unequivocal chromogranin A positivity. In Case 4 (serous adenocarcinoma), PAX8 was positive, WT-1, ER, and p16 were negative, and p53 showed a wild-type pattern.

### 3.3. HPV Status and IECC Classification

hrHPV DNA testing was performed in seven of ten patients (Table 3). DNA was detected in six tumors and not detected in one (the stage IIIC1p signet-ring cell adenocarcinoma); testing was unavailable in three (the sarcomatoid carcinoma, the stage IVB signet-ring cell adenocarcinoma, and the granulocytic sarcoma). p16 was positive in the two SCNECC (Case 7, focal/positive; Case 8, diffuse block-type positive) and in one serous carcinoma (Case 9, diffuse block-type positive), and negative in the serous Case 4, the clear cell carcinoma, and the glassy cell carcinoma.

Three tumors were classified as HPV-associated based on the combined p16 and hrHPV DNA findings: both SCNECC and one serous adenocarcinoma (Case 9). Two were HPV-independent: the sarcomatoid carcinoma and the hrHPV DNA-negative signet-ring cell adenocarcinoma. Three tumors were hrHPV DNA-positive but p16-negative: one serous adenocarcinoma (Case 4), the clear cell carcinoma, and the glassy cell carcinoma. These lacked the p16 marker of transcriptional activity, so we classified them as HPV-independent rather than HPV-associated; the viral DNA indicates the presence of virus without evidence of E6/E7-driven oncogenesis. One tumor was non-epithelial (granulocytic sarcoma), and one was unclassified because testing was unavailable (the stage IVB signet-ring cell adenocarcinoma). Systematic testing therefore revealed frequent discordance between HPV DNA and p16, rather than a simple reclassification of these histotypes as HPV-driven.

### 3.4. Treatment and Outcomes

Seven patients underwent primary surgery, followed by adjuvant CRT (n = 6) or chemotherapy alone (n = 1, stage IVB); three received non-surgical treatment (CRT alone, CRT with brachytherapy, and systemic chemotherapy for the granulocytic sarcoma). Margins were negative in five of seven operated patients; one SCNECC had parametrial involvement and one signet-ring cell tumor had peritoneal carcinomatosis (metastatic dissemination). Lymph node metastasis was present in three patients (two para-aortic, one pelvic). The treatment and outcomes are in Table 4.

**Figure 2:**
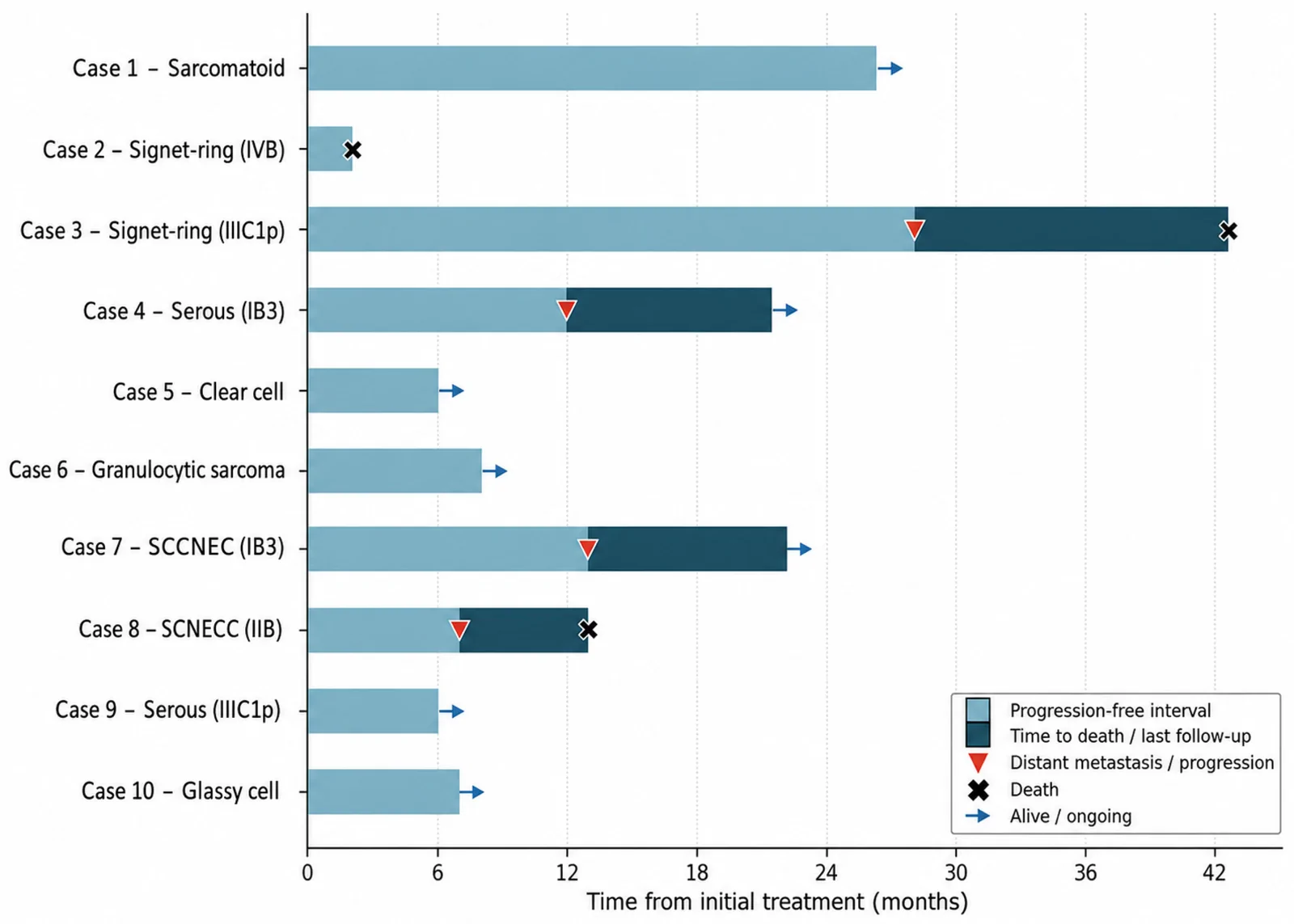
Swimmer plot of individual clinical courses (n = 10). Each bar begins at initial treatment; the light segment is the progression-free interval and the dark segment the subsequent time to death or last follow-up. Inverted triangles mark distant metastatic progression (no patient had isolated local cervical recurrence), crosses mark death, and arrowheads denote patients alive with ongoing follow-up. As fewer than half the patients had an event, the median PFS and OS were not reached; the plot is descriptive. SCNECC, small cell neuroendocrine carcinoma.

Over a median follow-up of 10.5 months (range 2–40), the median PFS and OS were not reached. No patient developed isolated local recurrence in the cervix or vaginal vault. All four progression events were distant metastases. Case 3 (signet-ring cell) developed pulmonary and peritoneal metastases at 28 months, received capecitabine–oxaliplatin, and died at 40 months. Case 4 (serous) developed pelvic and para-aortic nodal metastases at 12 months, received carboplatin–paclitaxel, and was alive at 22 months. Case 7 developed liver and lung metastases at 13 months, received cisplatin–etoposide, and was alive at 23 months. Case 8 (SCNECC) developed pelvic, para-aortic, and pulmonary metastases at 7 months, received cisplatin–etoposide, and died at 13 months. In total three patients died, and seven were alive at last contact, six of them without evidence of disease. The individual courses are shown in Figure 2.

## 4. Discussion

For each tumor in this series we recorded the antibodies applied, the mimics excluded, the HPV method used, and the basis for HPV-status assignment. Rare cervical histotypes are usually reported as isolated cases in which the work-up is compressed into a single line. Documenting it case by case lets the reader judge how securely each diagnosis was reached. Below we compare our findings with the published literature for each subtype. The reasoning that guided marker selection is summarized in Table 5.

### 4.1. HPV DNA Positivity Is Not Equivalent to HPV Association

Our most instructive finding concerns HPV-status assignment. Three tumors were hrHPV DNA-positive but p16-negative: one serous adenocarcinoma (Case 4), the clear cell carcinoma (Case 5), and the poorly differentiated adenosquamous (glassy cell) carcinoma (Case 10). This combination is informative rather than contradictory. Diffuse block-type p16 (CDKN2A) overexpression results from HPV E7-mediated inactivation of the retinoblastoma protein, and it is the validated surrogate for transcriptionally active hrHPV. The IECC and WHO frameworks therefore define HPV-association by morphology together with diffuse block-type p16 expression, not by the presence of viral DNA [1,5,8]. Any DNA-based assay, including the real-time PCR used here, can detect and type hrHPV DNA but cannot show whether the genome is integrated and transcribed or merely transient, episomal, or a bystander [3,4]. A DNA-positive but p16-negative result therefore means viral DNA is present without evidence of E6/E7-driven oncogenesis. Calling such tumors HPV-associated would overstate the viral contribution, so we classified them as HPV-independent.

This interpretation is not merely cautious; it is what the literature predicts for these histotypes. Clear cell carcinoma, the gastric-type and mucinous family, and many cervical serous carcinomas are prototypically HPV-independent. They are driven by TP53, PIK3CA, KRAS, STK11, and PTEN alterations rather than by HPV, and form a distinct, prognostically unfavorable molecular class [7,8,17]. Finding hrHPV DNA in a p16-negative clear cell or serous carcinoma is therefore expected background rather than evidence of viral causation. Our classification thus aligns the cohort with current WHO/IECC understanding rather than contradicting it. The practical message is that HPV DNA and p16 must be read together, that p16 with morphology governs the IECC category, and that E6/E7 mRNA or HPV RNA in situ hybridization should be used to resolve discordant cases where available [8].

### 4.2. Rarity of These Tumors and What This Series Adds

Each of these histotypes is very rare. SCNECC represents about 1–2% of cervical cancers, and the largest series include only a few hundred patients [10,11]. Primary cervical serous and clear cell carcinomas are reported mainly in small series and are increasingly reclassified under the IECC and WHO framework [5,17]. Primary cervical signet-ring cell carcinoma is described in only a few dozen cases [15,16], and glassy cell carcinoma accounts for well under 2% of cervical cancers [12,13]. Primary cervical granulocytic sarcoma is reported in only a handful of cases [18]. Against this background our contribution is diagnostic rather than statistical. We provide case-level virological and immunohistochemical documentation across seven rare histotypes seen consecutively at one center, we show frequent HPV DNA/p16 discordance, and we report a confirmed isolated primary cervical granulocytic sarcoma with bone marrow documentation. In the subsections that follow, our own observations are stated first and kept distinct from the cited literature.

### 4.3. Small Cell Neuroendocrine Carcinoma

Our two SCNECC cases show both the aggressiveness of this subtype and its diagnostic difficulty. Case 8 (stage IIB, diffuse block-type p16 positivity, hrHPV DNA-positive) had a fully supportive neuroendocrine profile: synaptophysin, chromogranin A, CD56, and INSM1 were positive, and Ki-67 was 60–70%. The course was aggressive, with pelvic, para-aortic, and pulmonary metastases and death at 13 months. Case 7 had an atypical profile: synaptophysin and CD56 were negative, chromogranin A positive, INSM1 equivocal, Ki-67 only 30–40%, and p63 and CD99 positive. Because SCNECC requires neuroendocrine confirmation [9,20], the reporting pathologist re-reviewed this case with control staining unchanged. The diagnosis was retained on the basis of characteristic small cell morphology and unequivocal chromogranin A positivity. Neuroendocrine marker expression in SCNECC is often heterogeneous, and synaptophysin or CD56 negativity occurs in otherwise typical tumors, where a single granular marker may be the only convincing positive [9,20]. Diffuse block-type p16 positivity with HPV18 further supports an HPV-associated neuroendocrine carcinoma [9]. We nevertheless acknowledge the atypical p63 and CD99 co-expression and the relatively low Ki-67, and we report this outcome with caution. In the literature, nodal and para-aortic disease predict poor outcome [21,22], and RB1 and PIK3CA are recurrent, potentially actionable drivers [23].

### 4.4. Clear Cell Carcinoma

Our clear cell carcinoma (Case 5) was hrHPV DNA-positive but p16-negative and is therefore classified here as HPV-independent rather than HPV-associated, in keeping with the predominantly HPV-independent nature of this histotype [17]. HNF1β, Napsin A, and PAX8 positivity supported the diagnosis (Table 5). The patient was LVSI-negative and remained recurrence-free at 6 months; whether HPV-independent clear cell carcinoma carries a different prognosis from any HPV-associated counterpart requires larger, genotype-resolved study [6].

### 4.5. Poorly Differentiated Adenosquamous (Glassy Cell) Carcinoma

In the 2020 WHO Classification, glassy cell carcinoma is no longer a distinct entity and is regarded as a poorly differentiated adenosquamous carcinoma [1]. We use this terminology but retain the older descriptor for continuity with the earlier literature. Our case (Case 10, LVSI-negative) was hrHPV DNA-positive but p16-negative and is therefore classified as HPV-independent. The patient received CRT plus interstitial brachytherapy and was recurrence-free at 7 months. CRT that includes brachytherapy achieves local control comparable to conventional adenosquamous carcinoma [24]. A meta-analysis reported about 55% 5-year survival, with limited benefit from radiotherapy alone [13], and HPV-negative cases are documented [12].

### 4.6. Sarcomatoid Carcinoma

Our sarcomatoid carcinoma (Case 1; HPV-independent) showed focal cytokeratin and EMA positivity within a spindled, pleomorphic background with mutant-pattern p53, distinguishing it from a true sarcoma in line with the current WHO criteria [1,14]. The patient had the longest disease-free interval in the series (26 months, alive). In the literature, stage is the dominant determinant of outcome, and surgically managed disease fares better than non-surgical disease [14].

### 4.7. Granulocytic Sarcoma

Primary cervical granulocytic (myeloid) sarcoma lies outside the HPV framework and is treated systemically rather than surgically, so its recognition is critical. Our patient (Case 6) had a myeloid immunophenotype (MPO, CD43, CD68, CD117, lysozyme; CD20/CD3 negative) and a normal bone marrow, establishing isolated primary extramedullary disease, a presentation reported in only a limited number of cervical cases [18]. AML-type cytarabine–daunorubicin induction was given; the patient was alive at 8 months, with continued marrow surveillance for progression to overt AML.

### 4.8. Signet-Ring Cell Adenocarcinoma

Our two signet-ring cell adenocarcinomas span the clinical spectrum. Case 2 (stage IVB) presented with peritoneal carcinomatosis and died at 2 months; hrHPV testing was unavailable, so the tumor is unclassified. A primary gastrointestinal tumor was excluded in this patient by colonoscopy, gastroscopy, and contrast-enhanced computed tomography of the thorax, abdomen, and pelvis, with dedicated evaluation of the pancreas and gastrointestinal organs. No separate primary lesion was found. These findings were reviewed with the reporting pathologist, and the pathology report concluded a primary cervical origin. The immunohistochemistry used to exclude a gastrointestinal primary (CK7/CK20, CDX2, SATB2, MUC5AC, MUC6, PAX8) was performed on the resected cervical tumor, not preoperatively. Peritoneal involvement was identified at surgery and confirmed histologically; it represents metastatic spread of the cervical primary and is not a criterion for establishing cervical origin [15,16]. Case 3 (stage IIIC1p, hrHPV DNA-negative, HPV-independent) remained progression-free for 28 months and then developed pulmonary and peritoneal metastases. Salvage capecitabine–oxaliplatin was given in keeping with the gastric-type biology of these tumors [25], and the patient died at 40 months.

### 4.9. Serous Adenocarcinoma

Our two serous adenocarcinomas differed in HPV status. Case 9 showed diffuse block-type p16 positivity with hrHPV DNA and met the criteria for HPV-association. Case 4 was hrHPV DNA-positive but p16-negative, with PAX8 positivity and negative WT-1, ER, and wild-type p53. Primary cervical serous carcinoma is a contested and now largely HPV-independent category under current WHO and ISGyP concepts [5,17]. The profile in Case 4 favors a primary cervical rather than ovarian tumor: diffuse WT-1 negativity argues against tubo-ovarian high-grade serous carcinoma, in which WT-1 is characteristically positive, while PAX8 positivity supports a Müllerian origin. A metastatic uterine or ovarian primary was also excluded clinically and radiologically, with no separate adnexal or endometrial mass. The wild-type p53 pattern shows that this tumor lacks the TP53 aberration typical of high-grade serous carcinoma. It is therefore better described as a p16-negative, HPV-independent Müllerian adenocarcinoma with serous-like features. We retain the serous designation for continuity, classify the tumor as HPV-independent, and present the case cautiously. It illustrates why p16 and morphology, not HPV DNA, should drive classification. Both patients had surgery and adjuvant CRT: Case 4 developed nodal metastases at 12 months and was alive at 22 months, and Case 9 was recurrence-free at 6 months.

### 4.10. Treatment and Emerging Therapies

Histology-adapted multimodal treatment is the current standard for rare cervical histologies [25]. We used concurrent cisplatin-based CRT across the epithelial subtypes, with subtype-specific systemic therapy. For context, a multicentric Turkish series reported 5-year OS above 80% for stage I–II conventional cervical cancer [26], far higher than expected for these rare, largely LVSI-positive tumors. Outcome expectations should therefore be calibrated to subtype. Because our series is small and heterogeneous, we draw no comparative or prognostic conclusions; the outcome data are descriptive only. Emerging options include CDK4/6 and PI3K-pathway inhibition in neuroendocrine tumors [23] and pembrolizumab for PD-L1-positive recurrent or metastatic cervical cancer [27,28], whose activity in rare histotypes needs dedicated study. Large cell neuroendocrine carcinoma is also more aggressive than the small cell variant [29].

## 5. Strengths and Limitations

This series has several limitations. The retrospective design, small size, and histologic heterogeneity prevent statistical or prognostic inference, so the outcome data are descriptive only. Two virological limitations also apply. First, hrHPV testing was unavailable in three patients. Second, E6/E7 mRNA testing and HPV RNA in situ hybridization were not performed, although the real-time PCR assay does distinguish HPV16 and HPV18. These techniques would show viral oncogene activity directly, so we relied on p16 with morphology to classify the DNA-positive, p16-negative tumors as HPV-independent. Two diagnoses (Cases 7 and 4) had atypical immunoprofiles; the reporting pathologist re-reviewed both with unchanged controls and confirmed them, and the supporting reasoning is given in the Discussion section. The strengths of this series are systematic case-level documentation of the diagnostic pathway across seven rare histotypes, uniform multidisciplinary management, pathological review by gynecologic pathologists, and a confirmed isolated primary cervical granulocytic sarcoma.

## 6. Conclusions

Rare cervical tumors require a structured diagnostic pathway. Subtype-directed immunohistochemistry confirms lineage and excludes a metastatic mimic, and hrHPV testing read together with p16 assigns HPV status. Our data show that HPV DNA alone does not establish HPV association: three DNA-positive tumors were p16-negative and are classified as HPV-independent. Morphology, HPV DNA, and p16 are therefore each insufficient on their own. Because these tumors behave aggressively and are treated according to histology, diagnostic precision directly informs treatment. We propose the algorithm in Figure 1 and the framework in Table 5 for routine use. Given the rarity and heterogeneity of these tumors, we also advocate centralized pathological review and prospective, genotype-resolved registries.

## Figures and Tables

**Table 1:** Clinicopathological characteristics of the ten cases (Results).

Case	Age	Cervical Cytology	Smoking	FIGO Stage	Histologic Subtype	Size (cm)	LVSI	Surgical Margin	ECOG PS
Case 1	60	Not performed	Smoker	IB2	Sarcomatoid carcinoma	3.1	Present	Negative	1
Case 2	54	Not performed	Non-smoker	IVB	Signet-ring cell adenocarcinoma	2.4	Present	Positive (peritoneal metastasis)	2
Case 3	37	Normal	Smoker	IIIC1p	Signet-ring cell adenocarcinoma	4.0	Present	Negative	0
Case 4	49	HSIL	Non-smoker	IB3	Serous adenocarcinoma	4.7	Present	Negative	1
Case 5	51	ASC-H	Non-smoker	IB3	Clear cell carcinoma	4.5	Absent	Negative	1
Case 6	82	Not performed	Smoker	IIB	Granulocytic sarcoma	6.0	Present	— (no surgery)	3
Case 7	52	AGC	Non-smoker	IB3	Small cell neuroendocrine carcinoma *	4.2	Present	Positive (parametrium)	2
Case 8	65	HSIL	Non-smoker	IIB	Small cell neuroendocrine carcinoma	7.0.	Present	— (no surgery)	3
Case 9	54	Invasive carcinoma	Non-smoker	IIIC1p	Serous adenocarcinoma	3.0	Present	Negative	0
Case 10	33	HSIL	Non-smoker	IIIA	Poorly differentiated adenosquamous (glassy cell)	4.0	Absent	— (no surgery)	0

AGC, atypical glandular cells; ASC-H, atypical squamous cells cannot exclude HSIL; ECOG PS, Eastern Cooperative Oncology Group Performance Status; HSIL, high-grade squamous intraepithelial lesion; LVSI, lymphovascular space invasion. * The diagnosis of Case 7 is discussed in Section 4.3 and was re-reviewed owing to an atypical immunoprofile. Virological data are in Table 3.

**Table 2:** Immunohistochemical results for each case (Results).

Case	Histologic Subtype	Immunohistochemistry (+, Positive; −, Negative; ±, Focal/Weak; NP, Not Performed)
Case 1	Sarcomatoid carcinoma	Cytokeratin (+), p53 (+, mutant pattern), vimentin (+), EMA (+)
Case 2	Signet-ring cell adenocarcinoma	CK7 (+), CK20 (+), CDX2 (−), SATB2 (−), PAX8 (−), MUC5AC (+), MUC6 (−), p16 (+)
Case 3	Signet-ring cell adenocarcinoma	CK7 (+), CK20 (±), CDX2 (−), SATB2 (−), PAX8 (+), MUC5AC (+), MUC6 (−), p16 (+)
Case 4	Serous adenocarcinoma	PAX8 (+), WT-1 (−), p53 (−, wild-type), ER (−), p16 (−)
Case 5	Clear cell carcinoma	HNF1β (+), Napsin A (+), PAX8 (+), p16 (−)
Case 6	Granulocytic (myeloid) sarcoma	MPO (+), CD43 (+), CD68 (+), CD117 (+), lysozyme (+), CD34 (NP), CD20 (−), CD3 (−)
Case 7	Small cell neuroendocrine carcinoma *	Synaptophysin (−), chromogranin A (+), CD56 (−), INSM1 (±), Ki-67 (30–40%), p16 (+), CD99 (+), p63 (+)
Case 8	Small cell neuroendocrine carcinoma	Synaptophysin (+), chromogranin A (+), CD56 (+), INSM1 (+), Ki-67 (60–70%), p16 (+)
Case 9	Serous adenocarcinoma	PAX8 (+), WT-1 (−), p53 (+, mutant pattern), ER (−), p16 (+)
Case 10	Poorly differentiated adenosquamous (glassy cell)	p16 (−), cytokeratin (+), mucin/PAS (+)

* Case 7 shows an atypical immunoprofile (synaptophysin/CD56 negative, INSM1 equivocal, comparatively low Ki-67, p63/CD99 positive) and was re-reviewed; see Section 4.3. Antibody clones, vendors, and dilutions are in Supplementary Table S2. EMA, epithelial membrane antigen; PAS, periodic acid–Schiff.

**Table 3:** Case-level HPV testing, p16 expression, and HPV-status assignment (Results).

Case	Histologic Subtype	Specimen	hrHPV Assay	hrHPV DNA	Genotype	p16 IHC	HPV Status
Case 1	Sarcomatoid carcinoma	Tumor tissue	Not tested	Not tested	—	Focal	HPV-independent
Case 2	Signet-ring cell adenocarcinoma	Tumor tissue	Not tested	Not tested	—	Block-type positive	Unclassified (not tested)
Case 3	Signet-ring cell adenocarcinoma	Tumor tissue	Qiagen real-time PCR †	Not detected	—	Block-type positive	HPV-independent (DNA−)
Case 4	Serous adenocarcinoma	Tumor tissue	Qiagen real-time PCR †	Detected	HPV18	Negative	HPV-independent (DNA+/p16−)
Case 5	Clear cell carcinoma	Tumor tissue	Qiagen real-time PCR †	Detected	HPV16	Negative	HPV-independent (DNA+/p16−)
Case 6	Granulocytic sarcoma	Tumor tissue	Not tested	Not tested	—	Negative	Non-epithelial
Case 7	Small cell neuroendocrine carcinoma *	Tumor tissue	Qiagen real-time PCR †	Detected	HPV18	Focal/positive	HPV-associated
Case 8	Small cell neuroendocrine carcinoma	Tumor tissue	Qiagen real-time PCR †	Detected	HPV18	Block-type positive	HPV-associated
Case 9	Serous adenocarcinoma	Tumor tissue	Qiagen real-time PCR †	Detected	HPV16	Block-type positive	HPV-associated
Case 10	Poorly differentiated adenosquamous (glassy cell)	Tumor tissue	Qiagen real-time PCR †	Detected	HPV18	Negative	HPV-independent (DNA+/p16−)

HPV-status assignment: diffuse block-type p16 positivity with hrHPV DNA = HPV-associated; hrHPV DNA not detected = HPV-independent; hrHPV DNA-positive but p16-negative = HPV-independent (not HPV-associated), the DNA reflecting virus present without the p16 surrogate of transcriptional activity. † Qiagen real-time PCR assay that separately identifies HPV16 and HPV18 and reports the remaining high-risk types collectively as “other hrHPV”. * Case 7 diagnosis re-reviewed (Section 4.3). hrHPV, high-risk human papillomavirus; IHC, immunohistochemistry.

**Table 4:** Treatment and clinical outcomes (Results).

**Case**	**Treatment**	**Chemotherapy Regimen**	**PFS (mo)**	**OS (mo)**	**Progression**	**Site of Metastasis/Progression**	**Salvage Therapy**	**Status**
Case 1	Surgery + CRT	Cisplatin (concurrent)	26	26	No	—	—	Alive
Case 2	Surgery + CT	Carboplatin + paclitaxel + bevacizumab	2	2	No	—	—	Dead
Case 3	Surgery + CRT	Cisplatin (concurrent)	28	40	Yes	Lung, peritoneal (metastases)	Capecitabine + oxaliplatin	Dead
Case 4	Surgery + CRT	Cisplatin (concurrent)	12	22	Yes	Pelvic and para-aortic nodal metastases	Carboplatin + paclitaxel	Alive
Case 5	Surgery + CRT	Cisplatin (concurrent)	6	6	No	—	—	Alive
Case 6	Chemotherapy	Cytarabine + daunorubicin	8	8	No	—	—	Alive
Case 7	Surgery + CRT	Cisplatin (concurrent)	13	23	Yes	Liver, lung (metastases)	Cisplatin + etoposide	Alive
Case 8	CRT	Cisplatin (concurrent)	7	13	Yes	Pelvis, para-aortic nodes, lung (metastases)	Cisplatin + etoposide	Dead
Case 9	Surgery + CRT	Cisplatin (concurrent)	6	6	No	—	—	Alive
Case 10	CRT + interstitial BRT	Cisplatin (concurrent)	7	7	No	—	—	Alive

All progression events were distant metastases; no patient had isolated local cervical recurrence. Bone marrow biopsy in Case 6 was normal, confirming isolated primary extramedullary myeloid sarcoma. Median PFS and OS were not reached (median follow-up of 10.5 months, range of 2–40). BRT, brachytherapy; CRT, chemoradiotherapy; CT, chemotherapy; mo, months; OS, overall survival; PFS, progression-free survival.

**Table 5:** Immunohistochemical framework for the differential diagnosis of rare cervical tumors (Discussion).

**Histotype**	**Characteristic/Positive Markers**	**Negative/Excluding Markers**	**Key Diagnostic Consideration**
Small cell neuroendocrine carcinoma	Synaptophysin, chromogranin A, CD56, INSM1; high Ki-67; p16 block-type positive	MPO, CD45 (LCA), p40, CK5/6, Napsin A, HNF-1β, S100/SOX10 (negative)	Confirm neuroendocrine lineage by IHC; morphology alone is unreliable [9,20]
Signet-ring cell adenocarcinoma	CK7, PAX8; p16 if HPV-associated	CDX2, SATB2, CK20, MUC5AC/MUC6 favor GI/gastric-type origin	Exclude gastrointestinal metastasis before a primary cervical diagnosis [15,16]
Serous adenocarcinoma	PAX8, WT-1, aberrant p53; ER variable	Napsin A, HNF-1β, MUC6, GATA3, CD10 (negative)	Exclude uterine/ovarian serous carcinoma; largely HPV-independent [5,17]
Clear cell carcinoma	HNF1β, Napsin A, PAX8	p16 (block-negative/patchy), ER, PR, MUC6, GATA3, CD10 (negative)	Predominantly HPV-independent [17]
Poorly differentiated adenosquamous (glassy cell)	p16 if HPV-associated; cytokeratin, mucin	Napsin A, HNF-1β, WT1, synaptophysin, chromogranin, INSM1 (negative)	No longer a distinct WHO entity; HPV-negative cases occur [12,13]
Sarcomatoid carcinoma	Focal cytokeratin/EMA within spindled/pleomorphic areas; p53	Desmin, myogenin, S100, SOX10, CD45, MPO, neuroendocrine markers (negative)	Focal epithelial differentiation distinguishes it from a true sarcoma; HPV-independent [14]
Granulocytic (myeloid) sarcoma	MPO, CD43, CD68, CD117, lysozyme, CD34	CD20, CD3, PAX5 (exclude lymphoma)	Bone marrow biopsy to confirm isolated disease; AML-based therapy [18]

General diagnostic reference synthesized from the cited literature; stains performed in the present cases are in Table 2. CK, cytokeratin; ER, estrogen receptor; GI, gastrointestinal; IHC, immunohistochemistry; LCA, leukocyte common antigen.

## Data Availability

De-identified data supporting the findings are available from the corresponding author upon reasonable request, subject to Institutional Ethics Committee approval and applicable data-protection regulations.

## Supplementary Materials

The following supporting information can be downloaded at: https://www.mdpi.com/article/10.3390/pathogens15090907/s1. Table S1, De-identified clinicopathological characteristics of the ten cases; Table S2, Immunohistochemistry antibody clones, vendors, and dilutions.

## Abbreviations

AGC: atypical glandular cells; AML, acute myeloid leukemia; ASC-H, atypical squamous cells cannot exclude HSIL; CRT, chemoradiotherapy; ECOG PS, Eastern Cooperative Oncology Group Performance Status; FIGO, International Federation of Gynecology and Obstetrics; GI, gastrointestinal; hrHPV, high-risk human papillomavirus; HPVA, HPV-associated; HPVI, HPV-independent; HSIL, high-grade squamous intraepithelial lesion; IECC, International Endocervical Adenocarcinoma Criteria and Classification; LVSI, lymphovascular space invasion; NP, not performed; OS, overall survival; PFS, progression-free survival; SCNECC, small cell neuroendocrine carcinoma of the cervix.

## References

[1] Höhn A.K., Brambs C.E., Hiller G.G.R., May D., Schmoeckel E., Horn L.C. (2021). 2020 WHO Classification of Female Genital Tumors. Geburtshilfe Frauenheilkd..

[2] Lee Y., Bae H., Kim H.S. (2022). Endocervical Adenocarcinoma: Comprehensive Histological Review and Re-classification of 123 Consecutive Cases According to the Updated WHO Classification of Female Genital Tumors. Anticancer Res..

[3] Walboomers J.M., Jacobs M.V., Manos M.M., Bosch X.F., Kummer J.A., Shah K.V., Snijders P.J.F., Peto J., Meijer C.J.L.M., Muñoz N. (1999). Human papillomavirus is a necessary cause of invasive cervical cancer worldwide. J. Pathol..

[4] Muñoz N., Bosch F.X., de Sanjosé S., Herrero R., Castellsagué X., Shah K.V., Snijders P.J.F., Meijer C.J.L.M. (2003). IARC Multicenter Cervical Cancer Study Group. Epidemiologic classification of human papillomavirus types associated with cervical cancer. N. Engl. J. Med..

[5] Stolnicu S., Barsan I., Hoang L., Patel P., Terinte C., Pesci A., Aviel-Ronen S., Kiyokawa T., Alvarado-Cabrero I., Pike M.C. (2018). International Endocervical Adenocarcinoma Criteria and Classification (IECC): A New Pathogenetic Classification for Invasive Adenocarcinomas of the Endocervix. Am. J. Surg. Pathol..

[6] Stolnicu S., Hoang L., Chiu D., Hanko-Bauer O., Terinte C., Pesci A., Aviel-Ronen S., Kiyokawa T., Alvarado-Cabrero I., Oliva E. (2019). Clinical Outcomes of HPV-associated and Unassociated Endocervical Adenocarcinomas Categorized by the IECC. Am. J. Surg. Pathol..

[7] Rodríguez-Carunchio L., Soveral I., Steenbergen R.D.M., Torné A., Martinez S., Fusté P., Pahisa J., Marimon L., Ordi J., del Pino M. (2015). HPV-negative carcinoma of the uterine cervix: A distinct type of cervical cancer with poor prognosis. BJOG Int. J. Obstet. Gynaecol..

[8] Giannella L., Grelloni C., Natalini L., Sartini G., Bordini M., Carpini G.D., Di Giuseppe J., Dugo E., Piva F., Ciavattini A. (2025). Molecular Features of HPV-Independent Cervical Cancers. Pathogens.

[9] Charbel A., Akiki G., Diwan S.S., Leung S.O.A., Kommoss F.K., Tessier-Cloutier B. (2026). Small cell neuroendocrine carcinoma of the cervix: Diagnostic challenges and emerging molecular insights. J. Pathol..

[10] Wang K.L., Chang T.C., Jung S.M., Chen C.-H., Cheng Y.-M., Wu H.-H., Liou W.-S., Hsu S.-T., Ou Y.-C., Yeh L.-S. (2012). Primary treatment and prognostic factors of small cell neuroendocrine carcinoma of the uterine cervix: A Taiwanese Gynecologic Oncology Group study. Eur. J. Cancer.

[11] Cohen J.G., Kapp D.S., Shin J.Y., Urban R., Sherman A.E., Chen L.-M., Osann K., Chan J.K. (2010). Small cell carcinoma of the cervix: Treatment and survival outcomes of 188 patients. Am. J. Obstet. Gynecol..

[12] Zolciak-Siwinska A., Jonska-Gmyrek J. (2014). Glassy cell carcinoma of the cervix: A literature review. Eur. J. Obstet. Gynecol. Reprod. Biol..

[13] Guitarte C., Alagkiozidis I., Mize B., Stevens E., Salame G., Lee Y.-C. (2014). Glassy cell carcinoma of the cervix: A systematic review and meta-analysis. Gynecol. Oncol..

[14] Majumder A., Rekhi B., Mittal N., Menon S., Deodhar K.K., Ghosh J., Maheshwari A. (2025). Carcinosarcoma of the cervix: Clinicopathological features of 17 tumors diagnosed at a Tertiary Cancer Referral Centre, India. Indian J. Pathol. Microbiol..

[15] Abad-Licham M., Cotrina C., Guerrero A., Gómez K., Astigueta J. (2024). Primary signet ring cell carcinoma of the cervix: Case report and literature review. Ecancermedicalscience.

[16] Sal V., Kahramanoglu I., Turan H., Tokgozoglu N., Bese T., Aydin O., Demirkiran F., Arvas M. (2016). Primary signet ring cell carcinoma of the cervix: A case report and review of the literature. Int. J. Surg. Case Rep..

[17] Giannella L., Di Giuseppe J., Delli Carpini G., Grelloni C., Fichera M., Sartini G., Caimmi S., Natalini L., Ciavattini A. (2022). HPV-Negative Adenocarcinomas of the Uterine Cervix: From Molecular Characterization to Clinical Implications. Int. J. Mol. Sci..

[18] Ye Z., Jiang Y. (2022). Granulocytic sarcoma of the cervix: A rare case report. Medicine.

[19] Bhatla N., Aoki D., Sharma D.N., Sankaranarayanan R. (2018). Cancer of the cervix uteri. Int. J. Gynaecol. Obstet..

[20] Du Y., Yang H., Feng Y., Wu Z., Li M., Zhu T., Zhuang Y. (2025). Diagnostic challenges in cervical small cell neuroendocrine carcinoma of a reproductive-age woman. Front. Oncol..

[21] Kobayashi M., Nakagawa S., Masuda T., Kakuda M., Hiramatsu K., Iwamiya T., Matsuzaki S., Hashimoto K., Ueda Y., Kodama M. (2025). Clinical and pathological characteristics and outcomes of small cell neuroendocrine carcinoma of the uterine cervix. Int. J. Gynecol. Cancer.

[22] Fernandes J., Hodgson A., Han K., Milosevic M., Lukovic J., Lheureux S., Ferguson S.E., Santiago A., Croke J. (2025). Clinical outcomes of neuroendocrine carcinoma of the cervix: Retrospective review from a large academic cancer centre. Gynecol. Oncol. Rep..

[23] Salvo G., Meyer L.A., Gonzales N.R., Frumovitz M., Hillman R.T. (2025). Neuroendocrine cervical carcinomas: Genomic insights, controversies in treatment strategies, and future directions: A NeCTuR study. Int. J. Gynecol. Cancer.

[24] Richard T.J., Fermín Y.E., Augusto O.C., Prado-Cucho S.L., Miranda-Narro A.I., Villoslada-Terrones V. (2025). Glassy cell carcinoma of the uterine cervix: A rare entity and literature review. Ecancermedicalscience.

[25] Arango-Bravo E.A., Galicia-Carmona T., Cetina-Pérez L., la Torre C.B.F.-D., Enríquez-Aceves M.I., García-Pacheco J.A., Gómez-García E.M. (2024). State of the art of cervical cancer treatment in rare histologies. Front. Oncol..

[26] Ozkaya Ucar Y., Aytekin O., Yalcin N., Oktar O., Yildirim H.E.K., Guner G.T., Gokkaya M., Unsal M., Tokalioglu A.A., Celik F. (2025). Survival Outcomes and Prognostic Factors for Patients in Early Stage Cervical Cancer: A Multicentric Study in Turkey. Diagnostics.

[27] Chung H.C., Ros W., Delord J.P., Perets R., Italiano A., Shapira-Frommer R., Manzuk L., Piha-Paul S., Xu L., Zeigenfuss S. (2019). Efficacy and Safety of Pembrolizumab in Previously Treated Advanced Cervical Cancer: Results From the Phase II KEYNOTE-158 Study. J. Clin. Oncol..

[28] Colombo N., Dubot C., Lorusso D., Caceres M.V., Hasegawa K., Shapira-Frommer R., Tewari K.S., Salman P., Hoyos Usta E., Yañez E. (2021). KEYNOTE-826 Investigators. Pembrolizumab for Persistent, Recurrent, or Metastatic Cervical Cancer. N. Engl. J. Med..

[29] Hu Y., Li D., Qiu L., Di W. (2026). Occult cervical large cell neuroendocrine carcinoma masquerading as adenocarcinoma in situ during pregnancy: A case report, literature review, and SEER analysis. BMC Pregnancy Childbirth.

