# Supplementary material for: Diagnosis of Rare Cervical Tumors: IECC Classification, HPV Status, and Subtype-Directed Immunohistochemistry, with Treatment Outcomes from a Tertiary Center
Source: Pathogens. 2026 Aug 28;15(9):907. doi: 10.3390/pathogens15090907 (PMC13610786; doi:10.3390/pathogens15090907)
Supplement: Supplementary file 1 [file pathogens-15-00907-s001.zip › pathogens-4499717-supplementary.pdf]

Supplementary Materials

Table S1. De-identified clinicopathological characteristics of the ten cases.

Case	Age	Cytology	HPV status	Smoking	FIGO stage	Histology	Size (cm)	LVSI	Margin	HPV category	ECOG
Case 1	60	Not performed	Not tested	Smoker	IB2	Sarcomatoid carcinoma	3.1	Present	Negative	HPV-independent	1
Case 2	54	Not performed	Not tested	Non-smoker	IVB	Signet-ring cell adenocarcinoma	2.4	Present	Positive (peritoneal metastasis)	Unclassified	2
Case 3	37	Normal	DNA not detected	Smoker	IIIC1p	Signet-ring cell adenocarcinoma	4.0	Present	Negative	HPV-independent	0
Case 4	49	HSIL	DNA+ / p16−	Non-smoker	IB3	Serous adenocarcinoma	4.7	Present	Negative	Indeterminate	1
Case 5	51	ASC-H	DNA+ / p16−	Non-smoker	IB3	Clear cell carcinoma	4.5	Absent	Negative	Indeterminate	1
Case 6	82	Not performed	Not tested	Smoker	IIB	Granulocytic sarcoma	6.0	Present	— (no surgery)	Non-epithelial	3
Case 7	52	AGC	DNA+ / p16+	Non-smoker	IB3	Small cell neuroendocrine carcinoma	4.2	Present	Positive (parametrium)	HPV-associated	2
Case 8	65	HSIL	DNA+ / p16+	Non-smoker	IIB	Small cell neuroendocrine carcinoma	7.0	Present	— (no surgery)	HPV-associated	3
Case 9	54	Invasive carcinoma	DNA+ / p16+	Non-smoker	IIIC1p	Serous adenocarcinoma	3.0	Present	Negative	HPV-associated	0
Case 10	33	HSIL	DNA+ / p16−	Non-smoker	IIIA	Poorly differentiated adenosquamous (glassy cell)	4.0	Absent	— (no surgery)	Indeterminate	0

No patient identifiers or initials are included. AGC, atypical glandular cells; ASC-H, atypical squamous cells cannot exclude HSIL; HSIL, high-grade squamous intraepithelial lesion; LVSI, lymphovascular space invasion; ECOG, Eastern Cooperative Oncology Group performance status.

Table S2. Immunohistochemistry — antibodies, clones, vendors, and dilutions.

Marker	Clone	Vendor (city, country)	Dilution	Retrieval Tamponade	Platform / detection
p16 (CDKN2A)	BC42	Biocare Medical Pacheco, CA, USA	1/100	Citrate	TALENT™ Automatic IHC Stainer/ iVision™ Poly-HRP Detection System
Synaptophysin	27G12	Biocare Medical Pacheco, CA, USA	1/100	EDTA	TALENT™ Automatic IHC Stainer/ iVision™ Poly-HRP Detection System
Chromogranin A	PHE5	Biocare Medical Pacheco, CA, USA	1/100	Citrate	TALENT™ Automatic IHC Stainer/ iVision™ Poly-HRP Detection System
CD56	BC56C06	Biocare Medical Pacheco, CA, USA	1/100	Citrate	TALENT™ Automatic IHC Stainer/ iVision™ Poly-HRP Detection System
INSM1	A8	Medaysis Reno, Nevada, USA	1/100	EDTA	TALENT™ Automatic IHC Stainer/ iVision™ Poly-HRP Detection System
Ki-67	MIB-1	Talent, Beijing, China	1/100	Citrate	TALENT™ Automatic IHC Stainer/ iVision™ Poly-HRP Detection System TALENT™ Automatic IHC Stainer/ iVision™ Poly-HRP Detection System
CK7	BC1	Biocare Medical Pacheco, CA, USA	1/100	Citrate	TALENT™ Automatic IHC Stainer/ iVision™ Poly-HRP Detection System
CK20	Ks20.8	Biocare Medical Pacheco, CA, USA	1/100	Citrate	TALENT™ Automatic IHC Stainer/ iVision™ Poly-HRP Detection System
CDX2	CDX2-88	Biocare Medical Pacheco, CA, USA	1/100	Citrate	TALENT™ Automatic IHC Stainer/ iVision™ Poly-HRP Detection System
SATB2	CL0323	Biocare Medical Pacheco, CA, USA	1/50	EDTA	TALENT™ Automatic IHC Stainer/ iVision™ Poly-HRP Detection System
PAX8	Bc12	Biocare Medical Pacheco, CA, USA	1/100	EDTA	TALENT™ Automatic IHC Stainer/ iVision™ Poly-HRP Detection System
MUC5AC	45M1	ScyTek Laboratories, Logan, USA	Ready to Use	Citrate	TALENT™ Automatic IHC Stainer/ iVision™ Poly-HRP Detection System

Marker	Clone	Vendor (city, country)	Dilution	Retrieval Tamponade	Platform / detection
MUC6	CLH5	ScyTek Laboratories, Logan, USA	1/100	Citrate	TALENT™ Automatic IHC Stainer/ iVision™ Poly-HRP Detection System
WT-1	rWT1/857	Biocare Medical Pacheco, CA, USA	1/100	Citrate	TALENT™ Automatic IHC Stainer/ iVision™ Poly-HRP Detection System
p53	DO-7	Biocare Medical Pacheco, CA, USA	1/100	Citrate	TALENT™ Automatic IHC Stainer/ iVision™ Poly-HRP Detection System
ER	SP1	Biocare Medical Pacheco, CA, USA	1/50	Citrate	TALENT™ Automatic IHC Stainer/ iVision™ Poly-HRP Detection System
HNF1β	EPR18644	Medaysis Reno, Nevada, USA	1/100	EDTA	TALENT™ Automatic IHC Stainer/ iVision™ Poly-HRP Detection System
Napsin A	TMU-Ad02	Biocare Medical Pacheco, CA, USA	1/100	Citrate	TALENT™ Automatic IHC Stainer/ iVision™ Poly-HRP Detection System
MPO	N/A	Biocare Medical Pacheco, CA, USA	Ready to Use	Citrate	TALENT™ Automatic IHC Stainer/ iVision™ Poly-HRP Detection System
CD43	DF-T1	Biocare Medical Pacheco, CA, USA	1/100	Citrate	TALENT™ Automatic IHC Stainer/ iVision™ Poly-HRP Detection System
CD68	KP1	Biocare Medical Pacheco, CA, USA	1/100	Citrate	TALENT™ Automatic IHC Stainer/ iVision™ Poly-HRP Detection System
CD117	EP10(previously known as Y145)	Biocare Medical Pacheco, CA, USA	1/100	Citrate	TALENT™ Automatic IHC Stainer/ iVision™ Poly-HRP Detection System
lysozyme	POLYCLONAL	ScyTek Laboratories, Logan, USA	Ready to Use	Citrate	TALENT™ Automatic IHC Stainer/ iVision™ Poly-HRP Detection System
CD34	QBEnd/10	Biocare Medical Pacheco, CA, USA	1/100	Citrate	TALENT™ Automatic IHC Stainer/ iVision™ Poly-HRP Detection System
CD20	L26	Biocare Medical Pacheco, CA, USA	1/100	Citrate	TALENT™ Automatic IHC Stainer/ iVision™ Poly-HRP Detection System
CD3	BC33	Biocare Medical Pacheco, CA, USA	1/100	Citrate	TALENT™ Automatic IHC Stainer/ iVision™ Poly-HRP Detection System

Marker	Clone	Vendor (city, country)	Dilution	Retrieval Tamponade	Platform / detection
p63	4A4	Biocare Medical Pacheco, CA, USA	1/100	Citrate	TALENT™ Automatic IHC Stainer/ iVision™ Poly-HRP Detection System
CD99	MIC/877	ScyTek Laboratories, Logan, USA	1/100	Citrate	TALENT™ Automatic IHC Stainer/ iVision™ Poly-HRP Detection System
EMA	E29	Biocare Medical Pacheco, CA, USA	1/100	Citrate	TALENT™ Automatic IHC Stainer/ iVision™ Poly-HRP Detection System
vimentin	V9	Biocare Medical Pacheco, CA, USA	1/100	Citrate	TALENT™ Automatic IHC Stainer/ iVision™ Poly-HRP Detection System
Pancytoceratin(PaNCK)	AE1/AE3	Biocare Medical Pacheco, CA, USA	1/100	Citrate	TALENT™ Automatic IHC Stainer/ iVision™ Poly-HRP Detection System
